# *Crude extracts from Allium cepa* skin and *Sorghum bicolor* seed can provide as non-toxic and eco-friendly cytoplasmic stains

**DOI:** 10.1016/j.plabm.2021.e00239

**Published:** 2021-06-05

**Authors:** Cecilia Krampah, Franklin Nyanzu, Abraham Quaye, Patrick Adu, Emmanuel Akomanin Asiamah, Benjamin Aboagye, David Larbi Simpong

**Affiliations:** a Department of Medical Laboratory Science, School of Allied Health Sciences, University of Ghana, Ghana; b Department of Medical Laboratory Science, School of Allied Health Sciences, University of Cape Coast, Ghana; c Department of Medical Laboratory Science, University of Allied Health Science, Ghana; d Forensic Science Department, University of Cape Coast, Ghana

**Keywords:** Natural dye, Stain, Cytoplasm, Cell, Nucleus, Extract

## Abstract

**Introduction:**

Staining is an important histological process; however, the use of non-toxic and environmentally friendly products is generally required. We explored the staining quality of two natural plants, *Allium cepa* skin and *Sorghum bicolor* seed extract on the cytoplasm.

**Materials and methods:**

Distilled water at 37 °C and 1% acid-ethanol were respectively used to extract the dyes from *Allium cepa* skin and *Sorghum bicolor seed*.

**Result:**

The application of these two dyes on rodent tissue showed an excellent cytoplasmic histomorphology.

**Conclusion:**

*Allium cepa* skin and *Sorghum bicolor* seed extracts are good cytoplasmic dyes when used as counterstain for haematoxylin.

## Highlights

• At acidic pH (3.5), *Allium cepa* skin extract stains cytoplasm and shows comparable result to eosin application.
• Similarly, at a pH of 7.0, crude extract from *Sorghum bicolor* seed stains the cytoplasm with clear cellular features.
• These extracts can serve as non-toxic and eco-friendly natural dyes for the demonstration of cellular and tissue morphology.

## 1 Introduction

Dyes are natural or synthetic products used to impact colour on a material or biological tissue. In histology laboratories, dyes (commonly termed stains) are used to impact colour on biological tissues to bring about contrast among cellular components. The application of dyes enables the cellular and tissue morphology to be apparent for evaluation. Dyes are used in forensic investigation, autopsy studies, clinical diagnosis, and in research [1]. Myriad dyes are available but, dyes of natural origin are preferred because it is non-toxic and eco-friendly [[2], [3], [4]]. Despite the potential threat of environmental pollutants from chemical-based synthetic dyes, the exploration of natural products as alternative for staining tissue has received little attention.

**Fig. 1:**
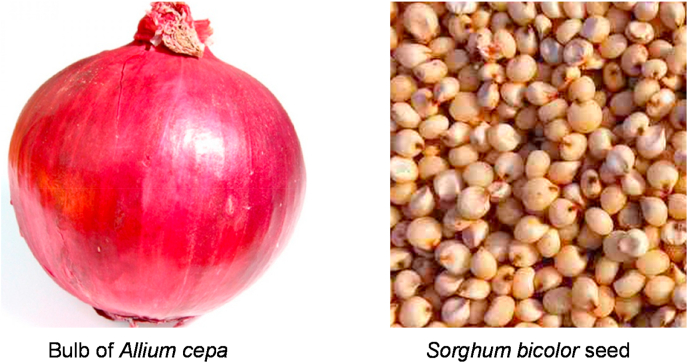
mage of *Allium cepa* and *Sorghum bicolor*.

This study explored the quality of several plant dyes on hepatocytes. It was observed that, crude extract from *Allium cepa* skin Fig. 1 and *Sorghum bicolor seed* Fig. 1 could distinguish the cytoplasm from the nucleus. Extract from any of these plants when used as counterstain for haematoxylin dye present an excellent cellular morphology. As cells are fundamental units of a tissue, the demonstration of cellular components indirectly evaluates tissue integrity. We maintained haematoxylin as the nuclear stain and used the crude extract from either *Allium cepa* skin or *Sorghum bicolor seed as* counterstain.

*Allium cepa* (red onion) is a member of the Liliaceae family, grown globally and generally used as food ingredient. Active constituents of *Allium cepa* include flavonoids, organosulfur compounds, and phenol components [5].

On the other hand, *Sorghum bicolor* is an important food crop found in Africa, south Asia, Central America. Extract from this plant is considered to possess diuretic, emollient remedy for cancer, epilepsy, gastric discomfort [6]. The plant is reach in phenolic compounds especially 3-deoxyanthocyanidins and tannins [7]. The adoption of these two plants was to evaluate their staining ability.

## 2 Materials and methods

### 2.1 Crude extraction of *Allium cepa*

Dry inedible papery skin of *Allium cepa* was peeled and collected for weighing. Ten grams of the dried papery *Allium cepa* skin was weighed using KERN EW620-3NM electronic balance and transferred into a clean dry beaker. 250 ml of distilled water was measured using volumetric flask and transferred to the beaker containing the dry inedible papery skin of the red Onion. The beaker was kept in a water bath at 37 °C for an hour. The solution obtained from the extraction was filtered with a Whatman's No1 filter paper fitted into a glass funnel to separate the onion skin residue from the extracted solution. The percolated solution obtained was kept at room temperature in a clean dry container and caped.

### 2.2 Crude extraction from the seed of *Sorghum bicolor*

Healthy brown dried *Sorghum bicolor* seeds were obtained and kept at room temperature until extraction. Two hundred and 50 g (250 g) of whole grain brown and red sorghum seeds were weighed using the KERN EW620-3NM electronic balance and transferred into a large mouth screw cap bottle. Acid ethanol (1%) was prepared by measuring 396 ml of absolute ethanol and 4 ml of concentrated hydrochloric acid using a measuring cylinder and gently adding the acid to the ethanol in a drop-wise fashion. The acidified ethanol was then poured into the bottle containing the weighed sorghum seeds, mixed and kept in a cool dark place for three (3) days. The leeched extract was filtered using high quality filter paper (Whatman's No.1) into a large beaker. The pH of the stain (reddish-brown coloured) was checked using the Mettler Toledo pH meter and was found to be 1.45. From the percolated extract, five (5) different pH (4.0, 5.5, 7.0, 9.0, and 10.5) were obtained by using HCl and aqueous potassium hydroxide solution to adjust the initial solution. Each container was labelled appropriately.

### 2.3 Tissue processing

The study involved imprinting control region (ICR) mice (n = 5) which were obtained from the Korle-Bu Animal House, euthanized in accordance with the guidelines and recommendations of Federation of European Laboratory Animal Science Association (FELASA) to harvest the liver. The harvested liver was kept in 10% neutral buffered formalin until needed.

Portions of the liver samples were cut using surgical blade into processing cassettes and labelled. The cassettes containing liver samples were processed using manual tissue processing method. Tissues were treated with 10% formalin for one (1) hour, dehydrated in ascending grades of ethanol (70%, 80% and 90%) for an hour each and three changes of absolute ethanol also for an hour each. The tissues were cleared in two changes of xylene for an hour each and infiltrated in two changes of molten paraffin wax (60 °C) for an hour each. Infiltrated tissues were embedded in paraffin wax to obtain tissue blocks. Five micrometre (5 μm) thick sections of tissues were cut using a rotary microtome and floated on water bath at 50 °C before being picked with a clean grease-free glass slide.

### 2.4 Staining

The conventional haematoxylin and eosin (H&E) staining technique (supplementary data table 3) was first used to stain sections of the liver tissue to serve as a control. Next, sections from the tissue were stained with the *Allium cepa* skin extract as described in supplementary data 1. Liver sections were first stained with haematoxylin. *Allium cepa* skin extract at a temperature of 45 °C was applied as a counterstain. The counterstain at 45 °C was applied in acidic (pH 3.5) medium in one set-up. In another set-up, the counterstain at 45 °C was applied at a basic (pH 8.0) medium.

In a separate set of staining, haematoxylin was used as primary dye to stain liver sections. As a counterstained, *Sorghum bicolor* seed extract at a pH of 7 was applied at room temperature (supplementary table 2). In another separate set-up, *Sorghum bicolor* seed extract at room temperature was applied at a pH of 5.5.

### 2.5 Scoring

Based on the staining appearance of the nucleus and the cytoplasm in each dye, a scoring grade of 0, 1, 2 and 3 were given. A score of 0 represented no staining of the nucleus or the cytoplasm. A score of 1 represented a weak staining of the nucleus or cytoplasm while a score of 2 represented moderate staining intensity and clarity. A score of 3 represents a strong and sharper staining intensity with microscopically clear cellular details.

## 3 Result

### 3.1 Staining with *Allium cepa* crude extract

**Fig. 2:**
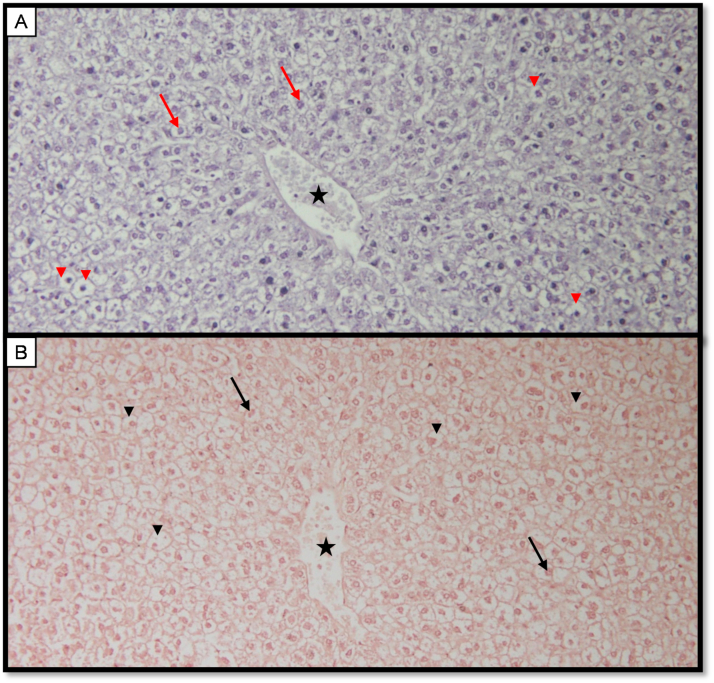
Staining characteristics of *Allium cepa* at different pH. **A** Photomicrograph of liver tissue stained with haematoxylin and counterstained with onion dye extract (pH 8.0: 45^O^C: 3mins). Scored 3+ demonstrating nucleus (arrow) and pale purple cytoplasm (arrowhead). The central vein in liver is shown by star **B** Photomicrograph of liver tissue stained with haematoxylin and counterstained with onion dye extract (pH 3.5: RT: 3mins). Scored 3+ demonstrating nucleus (arrow) and Pale to reddish brown cytoplasm (arrowhead). Magnification x200. (For interpretation of the references to color in this figure legend, the reader is referred to the Web version of this article.)

The results obtained when crude extract from the *Allium cepa* skin was used as a dye to stain liver tissue is shown in Fig. 2. Haematoxylin (nuclear stain) was used as primary dye and was counterstained using the crude extract from the onion. In Fig. 2A, the extract was applied at a pH of 8.0 and at a temperature of 45 °C for 3 min. Based on the result shown in Fig. 2A, a clear, sharp nuclear-cytoplasmic morphology was observed. Nuclei are shown by red arrow while cytoplasm is indicated by red arrowhead. A common feature of the liver tissue, the central vein (shown by a star) is also obvious.

In Fig. 2B, the *Allium cepa* skin extract was applied as a counterstain at a pH of 3.5 and at a room temperature (between 28 °C and 29 °C) for 3 min. Here, the cytoplasm is shown by pale reddish-brown colouration (black arrowhead). Also shown in Fig. 2B is the central vein (black star).

### 3.2 Staining reaction with *Sorghum bicolor* crude extract

**Fig. 3:**
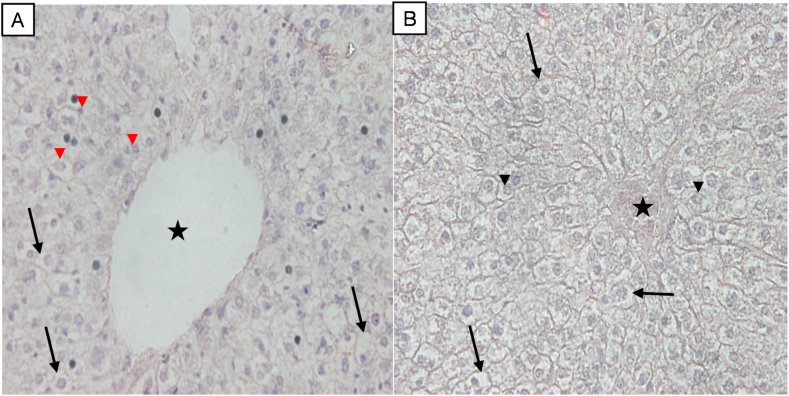
Staining characteristics of *Sorghum bicolor* at different pH. Liver tissue was stained initially with haematoxylin and counterstain with *Sorghum bicolor* crude extract at pH = 5.5 (**A**), and at a pH = 7.0 (**B**). Photomicrograph scored 2+ (**A**) and scored 3+ (**B**). Magnification x200.

Using crude extract from the *Sorghum bicolor,* liver tissue was first stained with haematoxylin and counterstained with *Sorghum bicolor* extract at pH 5.5 (Fig. 3A) as well as at pH 7.0 (Fig. 3B). Cellular morphology is clearly seen in both micrographs (A & B); however, photomicrograph B appears superior. Nuclei are shown by arrowhead while cytoplasm is indicated by an arrow. A distinct feature of the liver tissue, the central vein (indicated by a black star) is also apparent in both micrographs.

### 3.3 Liver tissue stained with haematoxylin and eosin

**Fig. 4:**
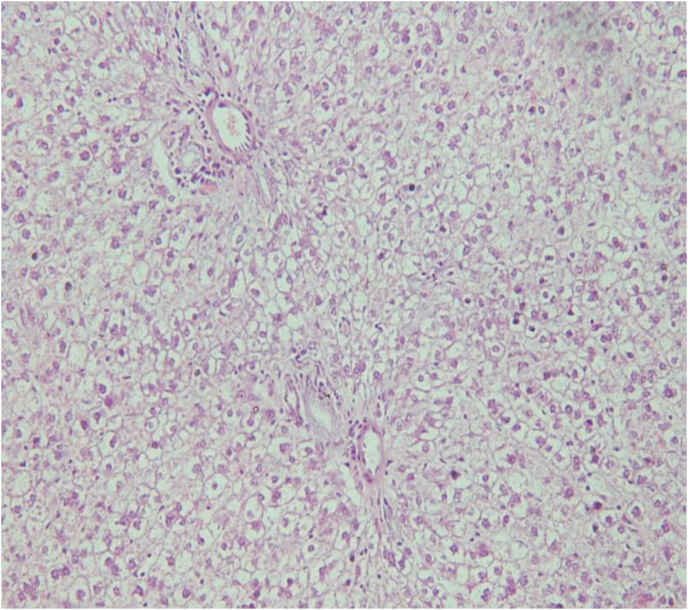
Liver tissue stained with haematoxylin and eosin. The photomicrograph shows a distinct feature (central vein) of the liver tissue. Magnification x200.

As a positive control, the conventional haematoxylin and eosin dyes were used to stain the liver tissue. From the micrograph (Fig. 4), the central vein is also shown.

## 4 Discussion

Tissue morphology is an important feature for the assessment of tissue integrity. To be discernible to the human eye, a tissue is stained with a suitable dye and viewed under the microscope. Dyes are obtained from either natural source or are synthetically produced. Both synthetic and natural dyes are useful for the evaluation of tissue architecture. However, the application of natural dyes is generally encouraged due to its environmentally friendliness, non-toxicity, and easy accessibility. As environmental issues are major concern globally, positive intervention or strategies aimed to mitigate the effects of ecological pollutants on the environment have become a major priority of the sustainable development goals (SDGs).

While synthetic dyes have helped in the assessment of tissue integrity, there is a concern regarding the waste management and disposal. Toxic chemical wastes are harmful to the aquatic ecosystem [8]. Toxic chemical waste from synthetic dyes could also be harmful to crops and livestock, with resultant harmful effect on human lives, directly or indirectly [9]. On the contrary, natural dyes are eco-friendly and are potentially important source of useful stains but are yet to be explored in the field of diagnostic histopathology [10]. The present study explored the feasibility of staining cellular cytoplasm using extract from two (2) local plants; *Sorghum bicolor and Allium cepa* independently.

In this experiment, crude extract from *Sorghum bicolor and Allium cepa* skin were used as counterstains to complement routine haematoxylin stain. Varying the pH and the temperature, crude extract from the skin of *Allium cepa* stained cellular cytoplasm pale to purple in basic medium (pH 8) at 45 °C (Fig. 2A). In acidic medium (pH 3.5), *Allium cepa* extract stained the cytoplasm pale to reddish brown at room temperature (Fig. 2B). Similarly, at room temperature, crude extract from *Sorghum bicolor* seed stained the cytoplasm purple at a pH of 7.0 and 5.5. Though the cytoplasm was stained at two different pH, result from pH of 7.0 (Fig. 2B) appears superior. The reason why this *Sorghum bicolor* seed extract can stain tissue at two different state (pH of 5.5 and 7.0) is unclear however, it could be speculated that the extract can stain tissue within a pH range of 5.5 and 7.0.

Taken together, we were able to stain mice liver tissue with crude extract from two natural products. The cellular membrane, cellular pattern and tissue architecture are clearly conspicuous in the tissue (Fig. 2, Fig. 3). A distinction between the nucleus and the cytoplasm can be appreciated. To evaluate tissue integrity, the arrangement of cells as well as the morphologic pattern of the nucleus to cytoplasm ratio is of major interest. Outcome from this study indicate the possibility of staining tissue with these two natural dyes. A prominent histological feature of the liver, the central canal (indicated by a star, Fig. 2, Fig. 3) is also apparent in both extracts. Based on the results from the present study, it is plausible to suggest that extract from the seed of *Sorghum bicolor* and the skin of *Allium cepa* are suitable dyes that can be used to demonstrate the cytoplasm. As extract from *Allium cepa* or *Sorghum bicolor* stain the cytoplasm of hepatocytes in acidic medium just like the acidic dye eosin, both extracts and eosin could have similar staining properties. It was noticed that staining intensity and differentiation were more linked to user preference like eosin usage. It is tempting to suggest that extract from *Allium cepa* or *Sorghum bicolor* demonstrate similar properties like eosin and it can therefore be used to stain the cytoplasm. As a positive control, the traditional method that employs haematoxylin and eosin was used to stain liver tissue and the result was comparable to that obtained using our local extract. If further evaluation and validation are performed, extract from these local plants may serve as a surrogate counterstain for haematoxylin dye.

## 5 Conclusion

Crude extract from *Sorghum bicolor* seed and *Allium cepa* skin are good cytoplasmic stains. Both extracts used in acidic medium can serve as counterstain for haematoxylin in the histopathology laboratory.

## Declaration of competing interest

Authors have none to declare.
