# Supplementary material for: Crude extracts from Allium cepa skin and Sorghum bicolor seed can provide as non-toxic and eco-friendly cytoplasmic stains
Source: Pract Lab Med. 2021 Jun 5;26:e00239. doi: 10.1016/j.plabm.2021.e00239 (PMC8193152; doi:10.1016/j.plabm.2021.e00239)
Supplement: Multimedia component 1 [file mmc1.docx]

PLABM e00239

**SUPPLEMENTARY DATA**

**Table 1:** Staining procedure for the *Allium cepa* extract

| **Procedure** | **Time** |
| --- | --- |
| 1. Deparaffinization of sections in two changes of xylene | 5 minutes each |
| 1. Dip in absolute alcohol | 2 minutes |
| 1. Rehydration of tissue through decreasing grades of alcohol (90%, 80%, 50%) | 3 minutes each |
| 1. Running tap water | 2 minutes |
| 1. Stain in Harris haematoxylin | 3 minutes |
| 1. Wash in running tap water until section is blue | 5 minutes |
| 1. Differentiate in 1% acid alcohol | 5 minutes |
| 1. Wash in running tap water until section is blue | 5 minutes |
| 1. **Stain in *Allium cepa* extract** | **3 minutes** |
| 1. Wash in running tap water | 3 minutes |
| 1. Dehydrate through increasing grade of alcohol (50%, 70%, 90% & absolute) | 3 minutes each |
| 1. Clear in two changes of xylene | 2 minutes each |
| 1. Mount with DPX and apply glass coverslip | --------------------- |

**Table 2:** Staining procedure for the *Sorghum bicolor* crude extract

| **Procedure** | **Time** |
| --- | --- |
| 1. Deparaffinization of sections in two changes of xylene | 5 minutes each |
| 1. Dip in absolute alcohol | 2 minutes |
| 1. Rehydration of tissue through decreasing grades of alcohol (90%, 80%, 50%) | 3 minutes each |
| 1. Running tap water | 2 minutes |
| 1. Stain in Harris haematoxylin | 3 minutes |
| 1. Wash in running tap water until section is blue | 5 minutes |
| 1. Differentiate in 1% acid alcohol | 5 minutes |
| 1. Wash in running tap water until section is blue | 5 minutes |
| 1. **Stain in *Sorghum bicolor* crude extract** | **3 minutes** |
| 1. Wash in running tap water | 3 minutes |
| 1. Dehydrate through increasing grade of alcohol (50%, 70%, 90% & absolute) | 3 minutes each |
| 1. Clear in two changes of xylene | 2 minutes each |
| 1. Mount with DPX and apply glass coverslip | --------------------- |

**Table 3:** Staining procedure for conventional haematoxylin and eosin

| **Procedure** | **Time** |
| --- | --- |
| 1. Deparaffinization of sections in two changes of xylene | 5 minutes each |
| 1. Dip in absolute alcohol | 2 minutes |
| 1. Rehydration of tissue through decreasing grades of alcohol (90%, 80%, 50%) | 3 minutes each |
| 1. Running tap water | 2 minutes |
| 1. Stain in Harris haematoxylin | 3 minutes |
| 1. Wash in running tap water until section is blue | 5 minutes |
| 1. Differentiate in 1% acid alcohol | 5 minutes |
| 1. Wash in running tap water until section is blue | 5 minutes |
| 1. **Stain in 1% eosin Y** | **10 minutes** |
| 1. Wash in running tap water | 3 minutes |
| 1. Dehydrate through increasing grade of alcohol (50%, 70%, 90% & absolute) | 3 minutes each |
| 1. Clear in two changes of xylene | 2 minutes each |
| 1. Mount with DPX and apply glass coverslip | --------------------- |
